# AI Denoising Improves Image Quality and Radiological Workflows in Pediatric Ultra-Low-Dose Thorax Computed Tomography Scans

**DOI:** 10.3390/tomography8040140

**Published:** 2022-06-24

**Authors:** Andreas S. Brendlin, Ulrich Schmid, David Plajer, Maryanna Chaika, Markus Mader, Robin Wrazidlo, Simon Männlin, Jakob Spogis, Arne Estler, Michael Esser, Jürgen Schäfer, Saif Afat, Ilias Tsiflikas

**Affiliations:** Department of Diagnostic and Interventional Radiology, Eberhard-Karls University, D-72076 Tuebingen, Germany; ulrich.schmid@student.uni-tuebingen.de (U.S.); david.plajer@med.uni-tuebingen.de (D.P.); maryanna.chaika@med.uni-tuebingen.de (M.C.); markus.mader@med.uni-tuebingen.de (M.M.); robin.wrazidlo@med.uni-tuebingen.de (R.W.); simon.maennlin@med.uni-tuebingen.de (S.M.); jakob.spogis@med.uni-tuebingen.de (J.S.); arne.estler@med.uni-tuebingen.de (A.E.); michael.esser@med.uni-tuebingen.de (M.E.); juergen.schaefer@med.uni-tuebingen.de (J.S.); saif.afat@med.uni-tuebingen.de (S.A.); ilias.tsiflikas@med.uni-tuebingen.de (I.T.)

**Keywords:** pneumonia, computed tomography, AI (artificial intelligence), image quality enhancement

## Abstract

(1) This study evaluates the impact of an AI denoising algorithm on image quality, diagnostic accuracy, and radiological workflows in pediatric chest ultra-low-dose CT (ULDCT). (2) Methods: 100 consecutive pediatric thorax ULDCT were included and reconstructed using weighted filtered back projection (wFBP), iterative reconstruction (ADMIRE 2), and AI denoising (PixelShine). Place-consistent noise measurements were used to compare objective image quality. Eight blinded readers independently rated the subjective image quality on a Likert scale (1 = worst to 5 = best). Each reader wrote a semiquantitative report to evaluate disease severity using a severity score with six common pathologies. The time to diagnosis was measured for each reader to compare the possible workflow benefits. Properly corrected mixed-effects analysis with post-hoc subgroup tests were used. Spearman’s correlation coefficient measured inter-reader agreement for the subjective image quality analysis and the severity score sheets. (3) Results: The highest noise was measured for wFBP, followed by ADMIRE 2, and PixelShine (76.9 ± 9.62 vs. 43.4 ± 4.45 vs. 34.8 ± 3.27 HU; each *p* < 0.001). The highest subjective image quality was measured for PixelShine, followed by ADMIRE 2, and wFBP (4 (4–5) vs. 3 (4–5) vs. 3 (2–4), each *p* < 0.001) with good inter-rater agreement (r ≥ 0.790; *p* ≤ 0.001). In diagnostic accuracy analysis, there was a good inter-rater agreement between the severity scores (r ≥ 0.764; *p* < 0.001) without significant differences between severity score items per reconstruction mode (F (5.71; 566) = 0.792; *p* = 0.570). The shortest time to diagnosis was measured for the PixelShine datasets, followed by ADMIRE 2, and wFBP (2.28 ± 1.56 vs. 2.45 ± 1.90 vs. 2.66 ± 2.31 min; F (1.000; 99.00) = 268.1; *p* < 0.001). (4) Conclusions: AI denoising significantly improves image quality in pediatric thorax ULDCT without compromising the diagnostic confidence and reduces the time to diagnosis substantially.

## 1. Introduction

Computed Tomography (CT) of the chest is an important diagnostic tool in pediatric patients to rule out severe cases of pneumonia. The COVID-19 pandemic has underlined the usefulness of chest CT in this context [1]. CT may also help identify potentially critical emergencies in oncological cases, especially pneumonia after stem cell transplantations [2]. Furthermore, in some congenital diseases, regional structural lung damage may be visible in CT before symptom onset, allowing for preventative rather than reactive therapy [3]. Nonetheless, radiation exposure in pediatric patients causes concern for difficultly in predictable long-term harms, as multiple studies have described increased cancer risks in similar contexts [4,5,6]. Hence, many ultra-low-dose CT (ULDCT) protocols have been established in the last decade, prominently featuring spectral shaping [7]. Spectral shaping is a technique that typically employs extra tin filter layers in front of the x-ray tube, effectively absorbing low-energy photons [8]. Especially in pulmonary CT, shaping the spectrum in favor of higher energies is acceptable, as the contribution of low-energy photons to overall signal yield is negligible [9]. However, although being more dose-efficient, spectral shaping ULDCT protocols nonetheless have lower overall image quality than their standard counterparts due to higher noise [10]. Recently, AI-based denoising software solutions enabled image quality enhancement capabilities beyond the limits of conventional reconstruction methods [11]. Yet, these novel solutions come with novel challenges, particularly loss of spatial information and blurring [12]. Therefore, prominent review articles advise evaluating such algorithms strictly on a clinical use-case basis [13]. To the best of our knowledge, a thorough investigation of the potential effects of AI denoising in pediatric thorax ULDCT has not been attempted so far. Therefore, our goal was to evaluate the effects of AI denoising in this context. We hypothesized that image quality and workflows in pediatric thorax ULDCT might benefit from AI denoising.

## 2. Materials and Methods

### 2.1. Study Design, Population, and Radiation Dose

An a priori power analysis using the software solution G*Power (ver. 3.1.9.7, Franz Faul, University of Kiel, Kiel, Germany) determined the necessary sample size (*f* = 1.18, α = 0.05, 1-β = 0.95) to be 100 patients [14]. The institutional review board approved retrospective eligibility analysis and data collection of our centers’ in-patients for this single-center study’s purpose from 1 January 2021 to 1 January 2022 with a waiver for the need for informed consent (#167/2022BO2). Initially, we performed a database search (keywords: pneumonia AND pediatric AND computed tomography). If a patient had received multiple thorax ULDCT in the given timeframe, we only included the most recent and removed the others (“duplicates”). Further exclusion criteria were clinical indications other than (suspected) pneumonia, contrast-enhanced imaging, and image acquisition protocols other than ULDCT. From the thus enrolled 100 patients, we collected age and sex at the procedure time from their clinical reports. For radiation dose comparison, the patient’s mean CT dose index (CTDI_vol_ in mGy), the mean dose-length product (DLP in mGy*cm), and the mean SSDE (size-specific dose estimate in mGy, [15,16]) were extracted using the dose-management software DoseM ^®^ (Infinitt Europe GmbH, Frankfurt am Main, Germany).

### 2.2. Image Acquisition and Reconstruction Parameters

All CT examinations were non-contrast-enhanced and performed on the same third generation CT scanner (SOMATOM Force; Siemens Healthineers, Erlangen, Germany). Attenuation-based tube current modulation (CARE Dose4D, reference mAs 190) was activated for the examination. The tube voltage was set to Sn100 (single-source 100 kV with tin filter). Collimation was set to 0.6 × 192/128 mm, pitch was 2.8 (“Flash mode”), and gantry rotation time to 0.25 s. A medium-soft kernel (Br40d) was used to reconstruct all images. The CT datasets were reconstructed in an axial orientation with a slice thickness and an increment of 1 mm using weighted Filtered Back Projection (wFBP) and Advanced Modeled Iterative Reconstruction strength 2 (ADMIRE^®^, Siemens Healthineers, Erlangen, Germany), as is standard in our institute. Additionally, a novel AI-based postprocessing software solution (PixelShine^®^, AlgoMedica, Sunnyvale, CA, USA) was used to denoise the wFBP images, resulting in three datasets per patient.

### 2.3. Objective Image Quality

All corresponding series per patient (wFBP, ADMIRE 2, PixelShine) were loaded into the open-source ImageJ distribution FIJI (ver. 1.53 K, Wayne Rasband, National Institutes of Health-NIH, Maryland, MD, USA) [17]. The wFBP series were used to draw a total of 30 regions of interest (ROIs) per patient bilaterally in homogeneous paraspinal muscles (3 ROIs on each side in 5 consecutive slices, diameter ≥ 1 cm^2^). The program then conveyed those ROIs into each loaded series and performed place-consistent measurements of mean CT numbers in Hounsfield Units (HU) and their standard deviation (SD). The SD of HU was defined as image noise.

### 2.4. Subjective Image Quality

The patient datasets were anonymized and randomized by a group member otherwise not associated with this project. A total of 8 blinded readers with different experience levels in pediatric thorax ULDCT independently rated the subjective image quality (Rater 1 = medical student at the end of a 3 month internship, Rater 2–6 = radiology residents with 1, 2, 3, 4, and 5 years of experience, Rater 7–8 = radiology consultants with 6 and 7 years of experience). Subjective image quality was rated on a 5-point Likert scale (1 = poor, 2 = subpar, 3 = fair; 4 = good, 5 = excellent) according to the diagnostic requirements mentioned in the chapter “Chest, General” of the European Guidelines on Image Quality in Computed Tomography [18].

### 2.5. Diagnostic Accuracy

Each reader filled out a custom semiquantitative severity score report table during the subjective image quality analysis sessions. This score evaluates the subjective conspicuity (0 = no affection, 1 = 0–50% affection, 2 = 51–100% affection) of six common pneumonia-related pathologies (peribronchial cuffing, mucus plugging, ground glass opacities, cavities, consolidations, air trapping) in six pulmonary regions (right upper lobe, right middle lobe, right lower lobe, left upper lobe, lingula segment, and left lower lobe).

### 2.6. Time to Diagnosis

The time to diagnosis (until the severity score report table was finished) was measured for each reader to explore the potential effects of reconstruction mode on radiological workflows. In addition, we performed post-hoc subgroup analyses for professional experience levels to investigate potential dependencies.

### 2.7. Statistical Analysis

Statistical analysis and illustration were performed using GraphPad Prism version 9.3.1 for Windows (GraphPad Software, San Diego, CA, USA). Data distribution was tested using the Shapiro–Wilk test. Normally distributed variables were expressed as mean ± SD, and non-normally distributed variables as median and interquartile range (IQR). Data analysis ensued using mixed-effects models with Greenhouse–Geisser correction in case of violation of sphericity, and two-stage step-up correction after Benjamini, Krieger, and Yekutieli to counteract the type-1-error increase in post-hoc multiple comparisons. An adjusted *p*-value ≤ 0.05 indicated statistical significance. Spearman’s correlation coefficient (r) was used to quantify the inter-rater agreement of the subjective image quality analysis and the severity score points. Correlation coefficient values of 0–0.30 were interpreted as negligible, 0.31–0.50 as low, 0.51–0.70 as moderate, 0.71–0.90 as good, and 0.91–1.00 as excellent levels of agreement.

## 3. Results

### 3.1. Patient Population

**Figure 1:**
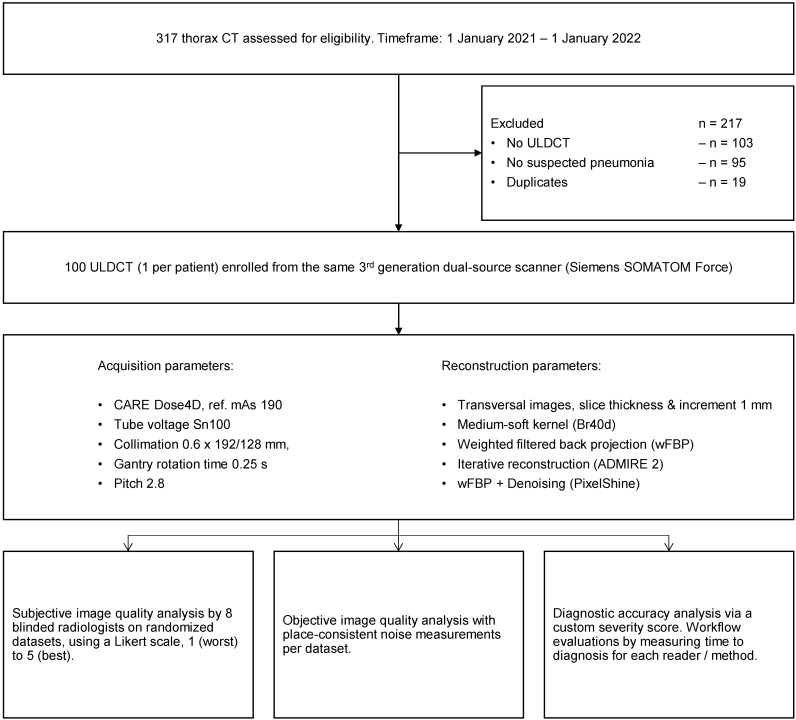
Patient enrollment and study workflow.

We evaluated 317 pediatric thorax ULDCT for eligibility, excluded 217, and included 100 examinations (1 examination per patient). From the included 100 examinations, we generated 300 datasets through reconstruction and postprocessing for further investigations. Figure 1 visualizes patient enrollment and the study workflow.

Our study cohort comprised 45 females and 55 males. At the time of the examination, the mean overall age was 10 ± 7 years (females: 10 ± 8 years; males 10 ± 6 years). Mean overall radiation exposure (SSDE) was 0.23 ± 0.09 mGy (females: 0.25 ± 0.11 mGy; males 0.22 ± 0.06 mGy). Mean overall CTDI_vol_ was 0.30 ± 0.16 mGy (females: 0.32 ± 0.20 mGy; males 0.28 ± 0.11 mGy) and mean overall DLP was 8.69 ± 5.54 mGy*cm (females: 8.80 ± 6.63 mGy*cm; males 0.60 ± 4.66 mGy*cm). In 60 patients (28 female), pneumonia screening CT was indicated due to oncological diseases, in 20 patients (8 female) due to infectious diseases, in 15 patients (8 female) due to congenital diseases, and in 5 patients (all male) in the course of surgical issues.

### 3.2. Objective Image Quality

**Figure 2:**
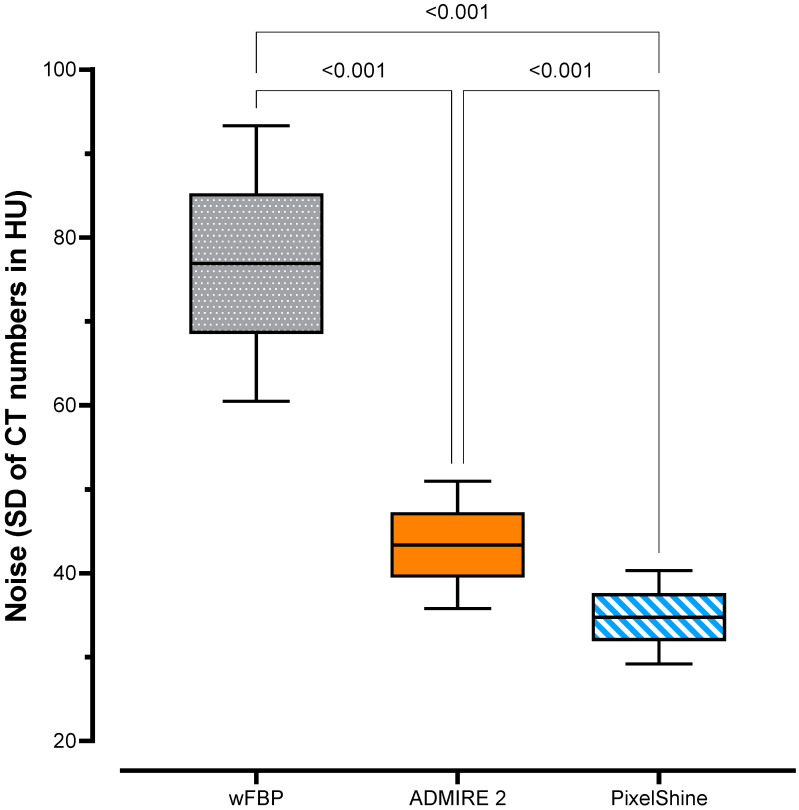
Noise measurements with pairwise comparisons.

The highest noise was measured for wFBP (76.9 ± 9.62 HU), followed by ADMIRE 2 (43.4 ± 4.45 HU) and PixelShine (34.8 ± 3.27 HU). Mixed-effects analysis showed significant differences between the groups (F (1.00; 99.0) = 4348; *p* < 0.001). Figure 2 graphs the data distribution of the noise measurements and corrected *p*-values of post hoc pairwise comparisons between each group.

### 3.3. Subjective Image Quality

In summary, subjective image quality was generally rated fair (median 3) for wFBP, good (median 4) for ADMIRE 2, and excellent (median 5) for PixelShine. There was a good inter-rater agreement for wFBP (r ≥ 0.764; *p* ≤ 0.001) and for PixelShine (r ≥ 0.790; *p* ≤ 0.001), and an excellent inter-rater agreement for ADMIRE 2 (r ≥ 0.908; *p* ≤ 0.001). See Table 1 for further details about subject image quality ratings and inter-rater-agreement.

**Figure 3:**
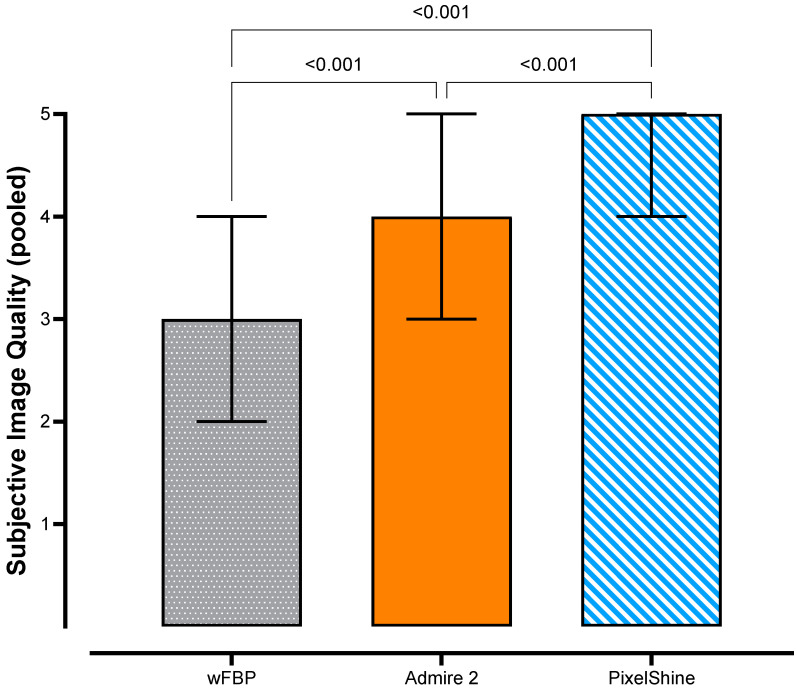
Pooled subjective image quality ratings with pairwise comparisons.

Overall, the highest pooled subjective image quality ratings were measured for PixelShine (4 (4–5)), followed by ADMIRE 2 (3 (4–5) and by wFBP (3 (2–4)), with statistically significant steps between each. Figure 3 shows the pooled subjective image quality rating distributions and corrected *p*-values of pairwise comparisons.

### 3.4. Diagnostic Accuracy

In wFBP reconstructions, the patients had a mean severity score of 10.00 ± 6.4 points, in ADMIRE 2 reconstructions 10.00 ± 6.40 points, and in PixelShine reconstructions 10.00 ± 6.63 points with good inter-rater agreement (wFBP r ≥ 0.764; ADMIRE 2 r ≥ 0.777; PixelShine r ≥ 0.826; each *p* < 0.001). See Table 2 for further details about severity score points and inter-rater-agreement.

**Figure 4:**
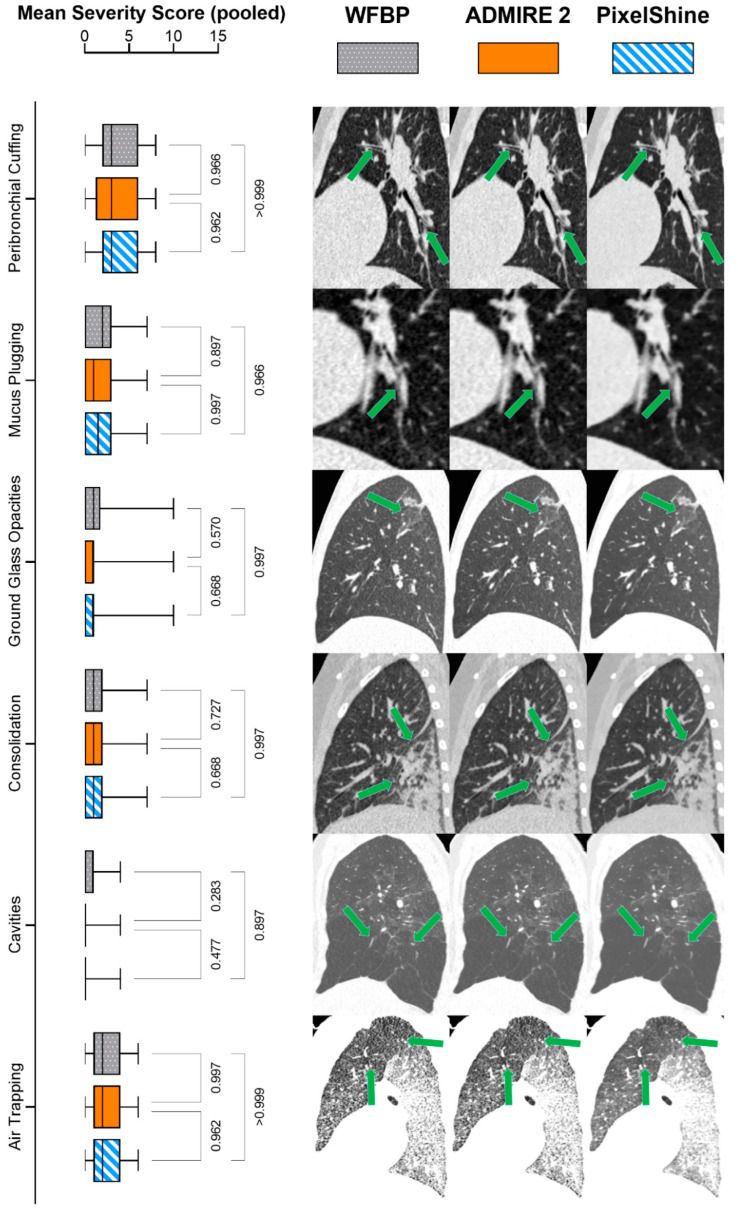
Mean severity score sums with pairwise comparisons and exemplary images. The green arrows highlight the investigated pathologies.

While there were significant interactions between the score items themselves (F (3.68; 364) = 45.7; *p* < 0.001), mixed-effects analysis did not reveal significant interactions for severity score items per reconstruction mode (F (5.71; 566) = 0.792; *p* = 0.570). Figure 4 shows post-hoc pairwise comparisons for the mean severity score sums per score item and series (wFBP, ADMIRE 2, PixelShine), as well as score-item-specific exemplary images.

### 3.5. Time to Diagnosis

**Figure 5:**
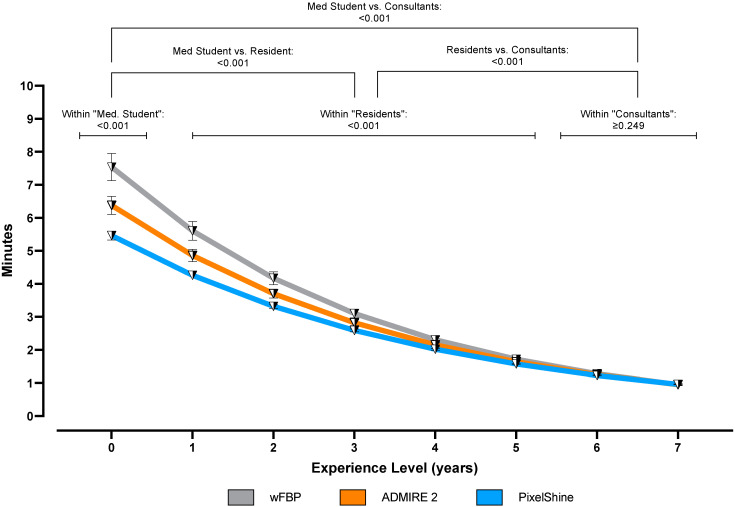
Time to diagnosis and pairwise comparisons.

Overall, time to diagnosis was highest in wFBP datasets (2.66 ± 2.31 min), followed by ADMIRE 2 (2.45 ± 1.90 min) and PixelShine (2.28 ± 1.56 min). Mixed-effects analysis showed significant interactions between these groups (F (1.000; 99.00) = 268.1; *p* < 0.001). Unsurprisingly, the post-hoc subgroup analyses revealed consultants to produce the fastest results (1.10 ± 0.01 min), followed by residents (3.05 ± 0.32 min) and medical students (6.46 ± 1.04 min). When examining the effect of reconstruction mode within these groups, it became clear that reports by medical students and residents were done significantly faster on the denoised datasets than on ADMIRE 2 and wFBP (each *p* < 0.001). For consultants, reconstruction mode did not affect the time to diagnosis (*p* ≥ 0.249). Figure 5 graphs the time to diagnosis per reconstruction mode in minutes as a function of experience levels in years.

## 4. Discussion

Although CT to rule out pneumonia is generally used with great reluctance in pediatric patients because of the difficultly predictable long-term effects, it is still a standard tool for time-efficient and comprehensive diagnoses. A promising technique to minimize potentially harmful radiation exposure in pediatric thorax ultra-low-dose CT protocols is to improve the dose-effectiveness by shaping the X-ray spectrum in favor of higher energy photons. However, the typical trade-off of such approaches is lower image quality than standard protocols due to higher image noise. This study examined the impact of a novel AI denoising algorithm on image quality, diagnostic accuracy, and radiological workflows. Multiple previous studies demonstrated impaired image quality in spectral shaping ULDCT protocols compared to full-spectrum protocols. For example, while Bodelle et al. described constant subjective image quality, image noise was higher in their spectral shaping study group [9]. Suntharalingam et al. reiterated these findings, describing objective image quality deterioration due to higher noise in their spectral shaping study group [19]. However, these studies focused on evaluating spectral shaping protocols in the first place, without additional postprocessing. Wetzl et al. reported significant image quality improvements in spectral shaping protocols using advanced iterative reconstruction [20]. In our study, AI denoising was capable of improving image quality beyond that of standard wFBP and iterative reconstruction datasets. Evaluating AI postprocessing has been the scope of several previous studies as well. For example, Kolb et al. pointed out significantly improved objective image quality in their denoised simulated low dose datasets. However, they also reported the subjective image quality of the original 100% dose images to still be significantly higher than that of the denoised images [21]. On the other hand, further studies described significantly improved subjective image quality of denoised datasets compared to regular reconstruction methods, with possible radiation dose reductions of up to 70% [11]. However, these studies focused on low-dose imaging via tube current-time product (mAs) reductions in full-spectrum protocols. AI image quality enhancement in spectral shaping protocols has not been attempted so far to the best of our knowledge, so the comparability of our results may be limited. Furthermore, minding the spectral distribution and the low acquisition parameters of the ULDCT protocol used in this study, the feasibility of similar dose reduction approaches remains the subject of further research. When using AI denoising, possible diagnostic confidence distortions through algorithmic misinterpretations are a commonly voiced concern [22]. However, Yang et al. reported improved lesion delineation when using CT AI denoising [23]. In our study, we measured retained diagnostic confidence on a radiological score-item level, proving the technique’s feasibility in this context. Several studies have evaluated the integrability of AI into radiological workflows and its potential benefits [24,25,26]. However, these applications generally increase the reporting time via supportive workflow improvements and computer-aided diagnoses. In our study, time to diagnosis increased significantly for medical students and residents when using the denoised datasets. However, the reporting time of consultant radiologists was not affected by reconstruction mode. As similar results have not been described so far, we can only hypothesize that denoising may help “declutter” medical images, which may be especially helpful for less-experienced radiologists. This study has several limitations. First, we used a retrospective study design. Although an a priori power analysis confirmed the validity of our results in this setup, any conclusions about the prospective clinical decision-making are nonetheless limited. Second, this study examined image quality metrics and diagnostic criteria related to suspected pneumonia in pediatric patients. As previous review articles have pointed out, AI denoising algorithms should be carefully evaluated on a use-case level. In our case, this especially pertains to potential incidental findings in thorax ULDCT, such as vascular malformations or potentially malignant lesions. As such analyses require thorough preparatory work to match normal prevalence distributions of potential incidental findings in pediatric ULDCT cohorts, additional studies are needed to validate the algorithm’s performance outside of the investigated medical issue. Lastly, this study was performed using a high-end third generation CT scanner, which might not be readily available at every site. Our results might therefore be specific to this setup.

## 5. Conclusions

AI denoising significantly improves image quality in pediatric thorax ULDCT without compromising the diagnostic confidence and reduces the time to diagnosis substantially.

## Figures and Tables

**Table 1:** Subjective image quality ratings and inter-rater-agreement.

		Rating	Spearman Correlation Coefficient							
		Median (IQR)								
			Reader 1	Reader 2	Reader 3	Reader 4	Reader 5	Reader 6	Reader 7	Reader 8
wFBP	Reader 1	3 (2–3)	1.000	0.843	0.975	0.834	0.962	0.823	0.953	0.813
	Reader 2	3 (2–4)	0.843	1.000	0.814	0.991	0.803	0.980	0.794	0.970
	Reader 3	3 (2–4)	0.975	0.814	1.000	0.805	0.988	0.794	0.979	0.784
	Reader 4	3 (2–4)	0.834	0.991	0.805	1.000	0.793	0.989	0.785	0.979
	Reader 5	3 (2–4)	0.962	0.803	0.988	0.793	1.000	0.782	0.991	0.772
	Reader 6	3 (2–4)	0.823	0.980	0.794	0.989	0.782	1.000	0.774	0.990
	Reader 7	3 (2–4)	0.953	0.794	0.979	0.785	0.991	0.774	1.000	0.764
	Reader 8	3 (2–4)	0.813	0.970	0.784	0.979	0.772	0.990	0.764	1.000
ADMIRE 2	Reader 1	4 (3–5)	1.000	0.970	0.944	0.933	0.922	0.917	0.912	0.908
	Reader 2	4 (3–5)	0.970	1.000	0.971	0.957	0.945	0.939	0.933	0.928
	Reader 3	4 (3–5)	0.944	0.971	1.000	0.985	0.971	0.964	0.957	0.951
	Reader 4	4 (3–5)	0.933	0.957	0.985	1.000	0.985	0.978	0.971	0.964
	Reader 5	4 (3–5)	0.922	0.945	0.971	0.985	1.000	0.992	0.985	0.978
	Reader 6	4 (3–5)	0.917	0.939	0.964	0.978	0.992	1.000	0.992	0.985
	Reader 7	4 (3–5)	0.912	0.933	0.957	0.971	0.985	0.992	1.000	0.992
	Reader 8	4 (3–5)	0.908	0.928	0.951	0.964	0.978	0.985	0.992	1.000
PixelShine	Reader 1	5 (4–5)	1.000	0.921	0.882	0.845	0.826	0.808	0.808	0.790
	Reader 2	5 (4–5)	0.921	1.000	0.958	0.918	0.898	0.878	0.878	0.858
	Reader 3	5 (4–5)	0.882	0.958	1.000	0.958	0.937	0.916	0.916	0.895
	Reader 4	5 (4–5)	0.845	0.918	0.958	1.000	0.978	0.956	0.956	0.935
	Reader 5	5 (4–5)	0.826	0.898	0.937	0.978	1.000	0.978	0.978	0.956
	Reader 6	5 (4–5)	0.808	0.878	0.916	0.956	0.978	1.000	1.000	0.977
	Reader 7	5 (4–5)	0.808	0.878	0.916	0.956	0.978	1.000	1.000	0.977
	Reader 8	5 (4–5)	0.790	0.858	0.895	0.935	0.956	0.977	0.977	1.000

wFBP = weighted filtered back-projection; ADMIRE 2 = Advanced Modeled Iterative Reconstruction strength 2; IQR = interquartile range.

**Table 2:** Severity score ratings and inter-rater-agreement.

		Severity Score	Spearman Correlation Coefficient							
		(Mean ± SD)								
			Reader 1	Reader 2	Reader 3	Reader 4	Reader 5	Reader 6	Reader 7	Reader 8
wFBP	Reader 1	11.90 ± 6.72	1.000	0.990	0.979	0.970	0.764	0.772	0.813	0.784
	Reader 2	11.70 ± 6.72	0.990	1.000	0.989	0.980	0.774	0.782	0.823	0.794
	Reader 3	11.60 ± 6.83	0.979	0.989	1.000	0.991	0.785	0.793	0.834	0.805
	Reader 4	11.50 ± 6.86	0.970	0.980	0.991	1.000	0.794	0.803	0.843	0.814
	Reader 5	9.35 ± 5.92	0.764	0.774	0.785	0.794	1.000	0.991	0.953	0.979
	Reader 6	9.15 ± 5.84	0.772	0.782	0.793	0.803	0.991	1.000	0.962	0.988
	Reader 7	9.03 ± 5.88	0.813	0.823	0.834	0.843	0.953	0.962	1.000	0.975
	Reader 8	9.03 ± 5.81	0.784	0.794	0.805	0.814	0.979	0.988	0.975	1.000
ADMIRE 2	Reader 1	11.60 ± 6.72	1.000	0.996	0.993	0.989	0.782	0.778	0.783	0.777
	Reader 2	11.50 ± 6.76	0.996	1.000	0.998	0.994	0.784	0.779	0.785	0.780
	Reader 3	11.40 ± 6.76	0.993	0.998	1.000	0.997	0.790	0.787	0.794	0.786
	Reader 4	11.30 ± 6.71	0.989	0.994	0.997	1.000	0.797	0.795	0.802	0.794
	Reader 5	9.17 ± 5.7	0.782	0.784	0.790	0.797	1.000	0.996	0.985	0.989
	Reader 6	9.12 ± 5.78	0.778	0.779	0.787	0.795	0.996	1.000	0.986	0.991
	Reader 7	9.00 ± 5.85	0.783	0.785	0.794	0.802	0.985	0.986	1.000	0.990
	Reader 8	9.02 ± 5.81	0.777	0.780	0.786	0.794	0.989	0.991	0.990	1.000
PixelShine	Reader 1	11.20 ± 6.45	1.000	0.998	0.996	0.996	0.830	0.831	0.861	0.826
	Reader 2	11.10 ± 6.49	0.998	1.000	0.997	0.996	0.831	0.832	0.864	0.827
	Reader 3	11.00 ± 6.43	0.996	0.997	1.000	1.000	0.845	0.845	0.878	0.840
	Reader 4	11.00 ± 6.45	0.996	0.996	1.000	1.000	0.846	0.846	0.879	0.841
	Reader 5	9.26 ± 5.93	0.830	0.831	0.845	0.846	1.000	0.999	0.989	0.998
	Reader 6	9.23 ± 5.92	0.831	0.832	0.845	0.846	0.999	1.000	0.990	0.999
	Reader 7	9.41 ± 5.93	0.861	0.864	0.878	0.879	0.989	0.990	1.000	0.988
	Reader 8	9.18 ± 5.90	0.826	0.827	0.840	0.841	0.998	0.999	0.988	1.000

wFBP = weighted filtered back-projection; ADMIRE 2 = Advanced Modeled Iterative Reconstruction strength 2; SD = standard deviation.

## Data Availability

Data is contained within the article.
